# Methyldopa-Induced Autoimmune Antibodies in Pregnant Women—Case Series and Literature Review

**DOI:** 10.3390/jcm15218741

**Published:** 2026-11-09

**Authors:** Wiktoria Grycuk, Magdalena Pach, Diana Massalska

**Affiliations:** 1 Warsaw Institute of Women’s Health, 00-189 Warsaw, Poland; wiktoriagrycuk@gmail.com (W.G.); magdalenapach97@gmail.com (M.P.); 2 Department of Gynecology, Obstetrics and Gynecological Endocrinology, Center of Postgraduate Medical Education, 01-813 Warsaw, Poland

**Keywords:** methyldopa, autoimmune antibodies, autoimmune hemolytic anemia, autoantibodies, red blood cells, pregnancy, hypertension, case report

## Abstract

**Background**: Methyldopa is widely used for the treatment of hypertension during pregnancy because of its established maternal and fetal safety profile. However, it can induce red blood cell autoantibodies, occasionally resulting in autoimmune hemolytic anemia. The aim of this study was to summarize current evidence on methyldopa-associated red blood cell autoantibody formation and autoimmune hemolytic anemia in pregnant women, based on a review of the available literature and observations from our center. **Methods**: A scoping review was conducted using the PubMed/MEDLINE, Web of Science, and Scopus databases to identify studies addressing autoimmune antibodies or hemolytic anemia associated with methyldopa therapy in pregnancy. Two independent reviewers screened titles and abstracts and assessed full-text articles for eligibility. Disagreements were resolved through discussion with a third reviewer. In addition, citation mapping was performed using the Litmaps tool (Litmaps Ltd.) to expand the set of relevant studies. In addition to the literature findings, two cases from our center, consistent with the scope of this review, were included in the study. **Results:** Four studies met the inclusion criteria, all of which were case-based. In addition, two cases from our center were included. The available data on six pregnancies suggest that methyldopa may induce the production of autoantibodies with variable clinical expression—most often asymptomatic, although in some cases hemolytic anemia may occur. These autoantibodies can cross the placenta. Neonatal outcomes are likewise heterogeneous, ranging from asymptomatic presentations to hemolysis with associated jaundice. **Conclusions**: Methyldopa-induced autoantibody formation in pregnancy is often a benign finding; however, it requires careful evaluation to exclude hemolytic anemia. Drug discontinuation appears effective in symptomatic patients, whereas management of asymptomatic cases is not well defined and should be individualized. Further studies are needed to clarify the incidence and guide clinical management.

## 1. Introduction

Methyldopa, a centrally acting α2-adrenergic receptor agonist, has been extensively used in the treatment of hypertension during pregnancy owing to its favorable safety profile for both mother and fetus [1,2]. Methyldopa is also one of the first drugs recognized to induce autoimmune antibodies [3]. Specifically, it has been implicated in the development of autoimmune hemolytic anemia as well as the production of red blood cell antibodies with a positive direct antiglobulin test (DAT, direct Coombs test) without evidence of hemolysis [4,5,6]. This entity is rarely encountered today due to declining use of the drug in the general population. However, the possibility of methyldopa-induced autoimmune complications remains clinically relevant, especially in pregnant women, where methyldopa is extensively used for the treatment of hypertension. The exact mechanism underlying methyldopa-induced antibody formation remains unknown; however, it probably differs from the mechanisms responsible for other drug-induced hemolytic anemias [4]. Rather than promoting the formation of antibodies against drug–red blood cell (RBC) complexes, methyldopa appears to stimulate the production of autoantibodies directed against antigens on the red cell membrane, most commonly within the Rh system [3,4]. The proposed explanation is that methyldopa may act as a hapten which interacts with the RBC membrane and alters the structure of red cell proteins. This may trigger an autoimmune response, leading to the production of antibodies that can also cross-react with normal RBC antigens [3]. These antibodies are predominantly warm-type IgG, do not activate complement, and, when pathogenic, result in extravascular hemolysis [4]. According to early studies conducted in the 1960s, approximately 10–20% of patients treated with methyldopa for several months develop red cell autoantibodies, while less than 1% of these patients present clinical features of autoimmune hemolytic anemia [5,6]. However, these estimates are based on historical data, and the current incidence of methyldopa-induced red cell autoantibodies, particularly among pregnant women, remains poorly defined, as contemporary studies specifically addressing this issue are lacking. The severity of hemolysis varies from mild to severe, and the onset can be delayed, ranging from months to even years after initiation of therapy [4,6]. Discontinuation of methyldopa generally leads to resolution of hemolysis, although the DAT may remain positive for longer periods [4].

Despite many years of clinical experience with methyldopa, data specifically addressing its immunologic effects in pregnancy remain scarce. Its potential to induce autoimmune antibodies warrants further investigations. Here, we present a series of cases of methyldopa-induced autoimmune antibodies in pregnant women and provide a review of the available literature.

## 2. Materials and Methods

The objective of this scoping review was to summarize the current knowledge on methyldopa-associated autoimmune antibody formation and autoimmune hemolytic anemia in pregnant women, based on a review of the available literature and observations from our center.

Ethical review and approval were waived for this study because it involved a retrospective analysis of clinical records and did not involve any intervention beyond routine patient care. The study was conducted in accordance with institutional requirements regarding research ethics. Written informed consent for publication of this case series was obtained from both patients.

### 2.1. Literature Search

A search was conducted in three electronic databases: PubMed/MEDLINE, Web of Science, and Scopus. The search strategy included all available articles up to September 2026, regardless of the publication date. Only studies involving human subjects and published in English were considered. This scoping review was conducted in accordance with the PRISMA extension for Scoping Reviews (PRISMA-ScR) Checklist [7] which is provided in the Supplementary Materials (Table S1).

The following combination of keywords was used: (“autoimmune antibodies” OR “direct antiglobulin test” OR “DAT” OR “Coombs test” OR “erythrocyte antibodies” OR “red blood cell antibodies” OR “hemolytic anemia” OR “AIHA”) AND (“methyldopa” OR “alpha-methyldopa”) AND “pregnancy”.

### 2.2. Eligibility Criteria

All types of published studies were eligible for inclusion, including case reports, case series, clinical studies, and review articles, provided they addressed autoimmune antibodies or autoimmune hemolytic anemia in the context of methyldopa therapy in pregnant women. Exclusion criteria included duplicate publications and studies for which the abstract or full text was not available. Articles published in languages other than English were excluded.

### 2.3. Study Selection

All retrieved records were imported into Zotero (Academic Referencing Software, Digital Scholar version 6.0.37), where the automatic duplicate identifier function was used to remove duplicates. When duplicates were removed, two independent reviewers (W.G. and M.P.) screened the titles and abstracts of the retrieved records for eligibility. Disagreements were resolved through discussion with a third reviewer (D.M.). Full texts of potentially relevant studies were reviewed for inclusion. In addition, citation mapping was performed using the Litmaps tool (Litmaps Ltd., version 2025-01-16, Wellington, New Zealand) to expand the set of relevant studies. Articles included after full-text screening were used as seed papers, and their citation networks were explored to detect related studies that may not have been captured through the database search. These records were screened according to the same eligibility criteria as studies identified through database searching. Any disagreements regarding eligibility were resolved through discussion and consensus with a third reviewer (D.M.). The study selection process was documented using a PRISMA flow diagram, presented in Figure 1.

In addition to the literature findings, two cases from our center, consistent with the scope of this review, were included.

## 3. Results

Six cases of methyldopa-associated autoimmune antibody formation and autoimmune hemolytic anemia in pregnant women were included in the study (four from the literature and two from our center). Summarized findings of the reported cases are described in Table 1.

### 3.1. Case 1

A 37-year-old primigravida at 22 weeks of gestation, following in vitro fertilization (IVF), was referred for hematologic consultation due to a positive direct antiglobulin test (DAT) identified during routine serum antibody screening. Her medical history was significant for pre-existing hypertension, insulin resistance, and hyperandrogenism. At 10 weeks of gestation, she was diagnosed with mild drug-induced hepatic injury, most likely associated with progesterone therapy during the IVF procedure. Biochemical abnormalities resolved rapidly after discontinuation of progesterone, and liver function tests remained within normal limits throughout the rest of the pregnancy. At the presentation, the patient was receiving methyldopa 250 mg twice daily. Hematologic evaluation confirmed a positive DAT with IgG autoantibodies bound to the patient’s red blood cells. A direct antiglobulin test was performed using the microcolumn agglutination method. In this method, a polyvalent antiglobulin reagent—a mixture of anti-IgG and anti-C3 antibodies—is routinely used. When added to red blood cells coated with IgG immunoglobulins and/or the C3 component, it causes them to agglutinate, resulting in a positive test result [12]. Warm-type IgG autoantibodies were detected in the patient’s serum, whereas the indirect antiglobulin test (IAT) was negative, indicating the absence of circulating IgG alloantibodies. Laboratory findings included mild normocytic anemia (hemoglobin 10.3 g/dL); however, there was no evidence of hemolysis: reticulocyte count, serum lactate dehydrogenase (LDH), haptoglobin, and bilirubin levels were all within normal ranges. Physical examination was unremarkable and did not reveal clinical features suggestive of hemolytic anemia. Considering the positive DAT and ongoing methyldopa therapy, drug-induced autoantibody formation was suspected. Methyldopa was therefore discontinued and replaced with metoprolol 25 mg twice daily. As pregnancy progressed, inadequate blood pressure control necessitated an increase in the metoprolol dose to 100 mg four times daily, with amlodipine 10 mg once daily subsequently added to the treatment regimen. On follow-up, hemoglobin levels remained stable, and no laboratory or clinical signs of hemolytic anemia were observed in either the patient or the fetus. At 35 weeks of gestation, due to the development of severe preeclampsia and oligohydramnios, the patient underwent cesarean section and delivered a healthy male neonate with an Apgar score of 10. On physical examination, the newborn’s skin was jaundiced. Laboratory tests showed a slightly elevated total bilirubin level with no indication for phototherapy; follow-up blood counts showed no anemia. As the child was asymptomatic, a DAT was not performed. Nine months after delivery the mother underwent a repeat direct antiglobulin test, which proved negative. Laboratory findings of the patient and the newborn are summarized in Table 2 and Table 3, respectively.

### 3.2. Case 2

A 42-year-old primigravida at 27 weeks of gestation, following IVF, was referred for hematologic evaluation after a routine laboratory workup revealed a positive direct antiglobulin test, indicating the presence of IgG autoantibodies coating the patient’s red blood cells. A DAT was performed using the microcolumn agglutination method. Warm-type autoantibodies were detected in the patient’s serum, while the indirect antiglobulin test (IAT) was negative. Her medical history was notable for chronic hypertension and hypothyroidism, for which she received levothyroxine 125 µg daily and methyldopa 500 mg four times daily. Subsequent laboratory investigations revealed features of mild hemolysis, including an increased reticulocyte count, reduced haptoglobin concentration, mildly elevated lactate dehydrogenase (LDH), and erythrocyte anisocytosis, with normal bilirubin concentrations. The patient also reported fatigue and dizziness, which, although nonspecific, could suggest an ongoing hemolytic process. In view of suspected methyldopa-induced autoimmune hemolysis, methyldopa was discontinued and replaced with amlodipine 10 mg once daily and labetalol 100 mg twice daily, resulting in adequate blood pressure control. Follow-up evaluations showed stable hemoglobin levels, which remained within normal range, and resolution of hemolytic markers. The symptoms previously reported by the patient also improved. At 38 weeks of gestation, the patient underwent an emergency cesarean section during the second stage of labor due to a non-reassuring fetal heart rate. A healthy male infant was delivered with an Apgar score of 10. No clinical or laboratory evidence of hemolysis was observed in the neonate; therefore, laboratory tests, including the direct antiglobulin test, were not carried out. At 12 months after delivery, the mother underwent a repeat direct antiglobulin test, which proved positive. Laboratory findings of the patient are summarized in Table 4.

In summary, among the six cases of methyldopa use during pregnancy identified in the literature and at our center, three patients developed anemia, data were unavailable for one patient, and hemoglobin levels remained within the normal range in the other two patients. Patients with anemia reported symptoms including fatigue, pallor, tachycardia, and dizziness. In two of the three patients with anemia, laboratory findings suggestive of hemolysis were observed, including an elevated reticulocyte count and reduced haptoglobin concentration. Three neonates developed jaundice, one of whom also showed laboratory evidence of hemolysis. Two neonates required phototherapy. These findings highlight the heterogeneity of methyldopa-associated clinical presentations and laboratory abnormalities, ranging from asymptomatic cases to clinically significant hemolysis accompanied by jaundice.

## 4. Discussion

Gestational hypertension affects an estimated 5–10% of all pregnancies, and its complications represent one of the leading causes of maternal and fetal mortality [13,14]. According to current guidelines, labetalol, nifedipine, and methyldopa are recommended as first-line antihypertensive agents during pregnancy [15,16]. Methyldopa remains an important treatment option because of its well-established safety profile for both mother and fetus [14]. However, its capacity to induce red blood cell autoantibodies may pose diagnostic and clinical challenges, particularly in the context of routine antenatal screening in pregnant women.

The prevalence of methyldopa-induced red blood cell autoantibody formation in pregnant patients remains unclear, as only a few cases have been reported to date. In the general population, approximately 10–20% of individuals treated with methyldopa develop RBC autoantibodies; however, fewer than 1% develop clinical symptoms of autoimmune hemolytic anemia [5,17]. As these data are primarily derived from the studies in the context of long-term therapy (months or years), it may be hypothesized that the incidence in pregnant patients is lower due to shorter time of exposure to drug, limited to the duration of pregnancy. Therefore, it is crucial to consider whether the mere presence of these antibodies constitutes sufficient grounds for discontinuing methyldopa therapy, particularly given that therapeutic options for managing hypertension during pregnancy are limited.

The two cases presented in the study illustrate two distinct methyldopa-associated serologic and clinical presentations, namely, isolated DAT positivity without evidence of hemolysis and mild autoimmune hemolytic anemia. In both patients, warm-type IgG autoantibodies were detected in the absence of circulating alloantibodies. Although less common, methyldopa has also been reported to induce both positive DAT and IAT [18].

From a clinical perspective, the detection of a positive DAT in a pregnant patient receiving methyldopa raises a diagnostic dilemma. Distinguishing drug-induced autoantibodies from clinically significant alloantibodies is essential, as the latter may have important implications for the fetus. The cases described above, and previous literature reports, support the concept that DAT positivity alone does not necessarily indicate active hemolysis and should be interpreted in the context of clinical and laboratory findings. Grigoriadis et al. reported a case of symptomatic hemolytic anemia in a pregnant woman which resolved within one month after discontinuation of the drug [8]. Similarly, Bujandric et al. reported symptomatic hemolytic anemia developing 14 weeks after initiation of methyldopa therapy, with complete resolution following withdrawal of the drug [9]. These observations are consistent with our findings, in which discontinuation of methyldopa resulted in resolution of hematologic abnormalities.

Another aspect worth considering is the potential passage of maternal autoantibodies through the placenta and their effect on the fetus. Available data suggest that methyldopa-induced IgG autoantibodies may cross the placenta and result in a positive DAT in the newborn; however, the clinical consequences appear to be variable. In our analysis, a positive DAT was detected in two of the six neonates. In the case reported by Bujandrić et al., despite autoimmune hemolytic anemia in the mother, no hemolytic disease was observed in the newborn, and the DAT result was negative [9]. Similarly, in a case presented by Grigoriadis et al., the newborn of a woman treated with methyldopa, who developed hemolytic anemia and a strongly positive DAT for IgG, remained clinically asymptomatic after birth [8]. In contrast, Valsami et al. described a DAT-positive neonate who developed hemolytic anemia and jaundice requiring phototherapy [11]. Likewise, Ozdemir et al. reported a case of a neonate born to a mother treated with methyldopa who presented with a strongly positive DAT and jaundice requiring phototherapy, despite the absence of laboratory evidence of hemolytic anemia [10]. These cases also highlight the complexity of interpreting such findings, as a positive DAT in the newborn may also result from alloimmune hemolytic disease, most commonly due to ABO or Rh incompatibility, or less frequently other blood group systems such as Kell, Duffy, or Kidd [19]. Furthermore, it should be emphasized that methyldopa is not the only pharmacological agent capable of inducing anti-erythrocyte antibodies. Similar associations have been documented for other commonly used medications, including penicillins and cephalosporins [20]. In the aforementioned cases, it remains unclear whether methyldopa was the sole inducing agent.

No clear consensus exists regarding the diagnostic approach or management of patients with suspected methyldopa-induced autoantibody formation. In patients with clinical or laboratory signs of hemolysis, discontinuation of methyldopa is generally associated with resolution of hemolysis within several weeks, as demonstrated in previously reported cases and in our own observations. In the two cases from our center, the introduction of alternative antihypertensive therapy led to normalization of hematologic abnormalities without adverse maternal or fetal effects. However, data regarding the management of asymptomatic patients with isolated DAT positivity remain scarce. Future guidelines should define what type of monitoring and at what frequency is sufficient to avoid premature withdrawal of the drug. Given the limited therapeutic options for hypertension in pregnancy, the decision to discontinue methyldopa should balance the potential risks associated with ongoing autoantibody production against the benefits of effective blood pressure control.

The available evidence regarding the occurrence of methyldopa-induced autoantibodies in pregnancy remains limited, consisting primarily of case-based reports, with substantial heterogeneity in clinical and immunohematological reporting and a lack of standardized diagnostic and follow-up approaches. These limitations preclude definitive conclusions regarding the incidence, clinical course, and optimal evaluation and management of methyldopa-induced autoantibodies in pregnancy. Further studies are needed to better characterize the incidence and clinical significance of this phenomenon and to establish evidence-based recommendations for its management.

## 5. Conclusions

In conclusion, methyldopa-induced autoantibody formation is a clinically relevant but often benign phenomenon in pregnancy. Given the high prevalence of hypertension in this population, awareness of this entity is essential in routine obstetric practice. While most cases remain asymptomatic, clinicians should remain vigilant for signs of hemolysis and consider drug discontinuation when clinically indicated. Available evidence supports methyldopa withdrawal and substitution with alternative antihypertensive therapy in symptomatic patients. In asymptomatic cases, however, a personalized approach is warranted, balancing potential risks against the benefits of continued blood pressure control.

Further studies are needed for definitive conclusions regarding optimal evaluation and management of methyldopa-induced autoantibodies in pregnancy.

## Figures and Tables

**Figure 1:**
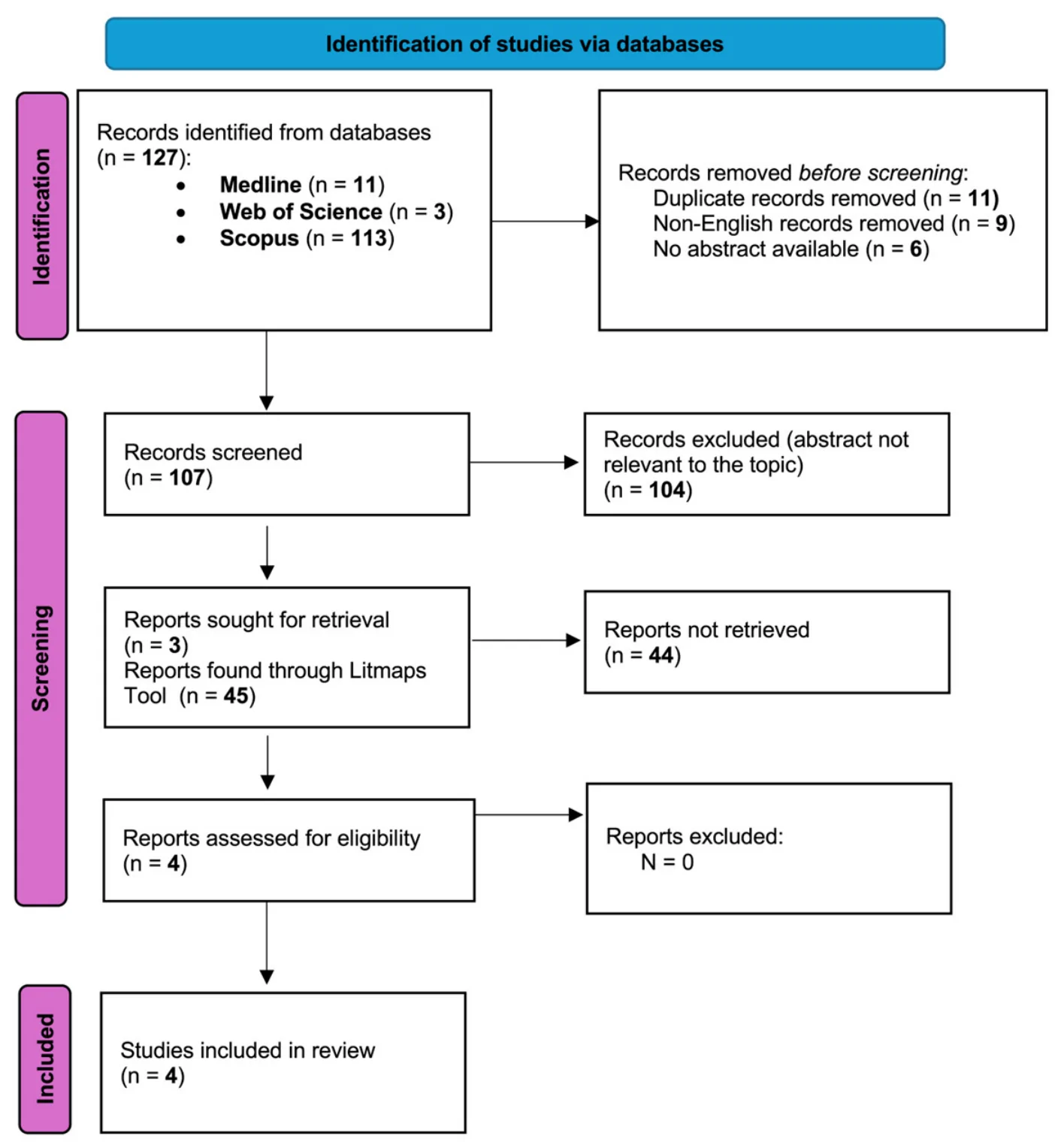
Flowchart for identifying eligible studies, adapted from Preferred Reporting Items for Systematic Reviews and Meta-Analyses (PRISMA) guidelines.

**Table 1:** Reported cases of autoimmune antibodies against red blood cells associated with methyldopa treatment in pregnant women.

Authors	Patient	Reason for Admission	Medical History	Methyldopa Dose	Findings at Presentation		Antibody Detection	Follow-Up
					Clinical Symptoms	Laboratory Findings		
Grigoriadis et al. [8]	36-year-old gravida 2 para 1, 37 + 6 weeks of gestation	Elective cesarean section	Mediterranean beta-thalassemia, hypertension	500 mg twice daily since the 24th week of gestation	Fatigue, skin pallor, tachycardia	Microcytic, hypochromic anemia (hemoglobin 8 g/dL)	Positive DAT	Hemoglobin levels returned to the pregestational levels (10 g/dL) one month after alpha-methyldopa discontinuation still with a positive DAT.
Bujandric and Grujic [9]	23-year-old gravida 1 para 1, 30 weeks of gestation	Anemia	Obesity, preeclampsia (hypertension, proteinuria, edema)	1500 mg daily since the 12th week of pregnancy	Fatigue, skin pallor	Anemia (hemoglobin 10.8 g/dL), low haptoglobin, high reticulocyte count (>10%)	Positive DAT; negative IAT; antibody identification was performed by the technique of agglutination with antihuman globulin at 37 degrees C	Anemia resolved after 3 weeks of methyldopa discontinuation.
Ozdemir et al. [10]	A female newborn whose mother took methyldopa in pregnancy	Jaundice after birth requiring phototherapy	Mother of a newborn: hypertension in pregnancy	Pregnant women: 500 mg twice daily	In newborn: jaundice, which occurred on the second day	Peak serum total bilirubin value was 18.7 mg/dL (indirect bilirubin value was 18 mg/dL); hemoglobin 16.2 g/dL, reticulocyte count 2%	In newborn: positive DAT; in mother: positive IAT (+), and DAT (+++)	No data available.
Valsami et al. [11]	A newborn with positive DAT result in cord blood sample, presumably secondary to maternal autoantibodies	Hemolytic disease of a newborn, jaundice requiring phototherapy	Mother of a newborn: hypertension in pregnancy	No data available	In newborn: signs of hemolysis, jaundice	No data available	In newborn: Positive DAT (+++); in mother: positive DAT (+++)	Good response to phototherapy; no further data available.
Case 1 (authors’ own observations)	37-year-old gravida 1, 22 weeks of gestation	Positive DAT identified during routine screening	Post-IVF, chronic hypertension, insulin resistance, hyperandrogenim, progesterone-induced mild hepatic injury	Methyldopa 250 mg twice daily, initiated prior to conception	No clinical symptoms reported	Normocytic anemia (hemoglobin 10.3 g/dL), no signs of hemolysis (reticulocyte count, LDH, haptoglobin, bilirubin levels within normal ranges)	Positive DAT, negative IAT, warm-type IgG autoantibodies detected in patient’s serum	Following discontinuation of methyldopa, hemoglobin levels remained stable, and no laboratory or clinical signs of hemolytic anemia were observed in either the patient, fetus or a newborn.
Case 2 (authors’ own observations)	42-year-old gravida 1, 27 weeks of gestation	Positive DAT identified during routine screening	Post-IVF, chronic hypertension, hypothyroidism,	Methyldopa 500 mg four times daily, initiated prior to conception	Fatigue, dizziness	Normal hemoglobin concentrations (11.6 g/dL); features of mild hemolysis (increased reticulocyte count, reduced haptoglobin concentration, mildly elevated LDH, erythrocyte anisocytosis); normal bilirubin concentrations	Positive DAT, negative IAT, warm-type IgG autoantibodies detected in patient’s serum	Following discontinuation of methyldopa, hemoglobin levels remained stable, hemolytic markers resolved; no laboratory or clinical signs of hemolytic anemia were observed in the patient, fetus or newborn.

Abbreviations: DAT—direct antiglobulin test; IAT—indirect antiglobulin test; LDH—lactate dehydrogenase.

**Table 2:** Patient’s laboratory findings following discontinuation of methyldopa (the drug was discontinued at 22 gestational weeks).

Laboratory Test [Normal Range]	22 Gestational Weeks	26 Gestational Weeks
Hemoglobin (g/dL) [10.8–15.0]	10.7	10.3
Reticulocyte count (%) [0.76–2.21]	1.27	2.33
Total bilirubin (mg/dL) [≤1.0]	0.21	0.15
LDH (U/l) [135–214]	138	128
Haptoglobin (g/L) [0.3–2.0]	0.8	0.8
Smear	RBC—no change	RBC—mild anisocytosis

**Table 3:** Laboratory findings in an infant born at 35 weeks’ gestation.

Laboratory Test [Normal Range]	1st Day After Birth	3rd Day After Birth	5th Day After Birth
Hemoglobin (g/dL) [13.0–24.0]	17.2	17.8	17.7
Direct bilirubin (mg/dL) [0–0.6]	N/A	0.37	0.46
Total bilirubin (mg/dL) [≤12.7]	N/A	9.18	12.84
ALT (U/L) [0–42]	N/A	17	N/A
AST (U/L) [24–100]	N/A	57	N/A

Abbreviations: N/A—not available.

**Table 4:** Patient’s laboratory findings before and after discontinuation of methyldopa at 27 gestational weeks.

Laboratory Test [Normal Range]	27 Gestational Weeks	31 Gestational Weeks
Hemoglobin (g/dL) [10.8–15.0]	11.6	11.9
Reticulocyte count (%) [0.76–2.21]	2.64	1.86
Direct bilirubin (mg/dL) [0.1–0.4]	0.13	0.16
LDH (U/L) [135–214]	222	230
Haptoglobin (g/L) [0.3–2.0]	0.4	0.35
Smear	RBC—anisocytosis	RBC—no change

## Data Availability

The datasets generated and analyzed in the current study are available from the corresponding author on reasonable request.

## Supplementary Materials

The following supporting information can be downloaded at: https://www.mdpi.com/article/10.3390/jcm15218741/s1, Table S1: Preferred Reporting Items for Systematic reviews and Meta-Analyses extension for Scoping Reviews (PRISMA-ScR) Checklist.
